# When mothers leave their mark

**DOI:** 10.7554/eLife.48899

**Published:** 2019-07-11

**Authors:** Raquel Barajas-Azpeleta, Kausik Si

**Affiliations:** Stowers Institute for Medical ResearchKansas CityUnited States

**Keywords:** epigenetic, transgenerational behavior, reproduction, inheritance, parasitoid wasp, NPF, *D. melanogaster*

## Abstract

Progeny can inherit parental experiences through altered brain chemistry.

**Related research article** Bozler J, Kacsoh BZ, Bosco G. 2019. Transgenerational inheritance of ethanol preference is caused by maternal NPF repression. *eLife* **8**:e45391. doi: 10.7554/eLife.45391

For many creatures, survival and reproduction depend on the ability to anticipate or quickly adapt to changes in the environment. For instance, parasitic wasps ensure that their hatchlings will have a fresh source of food by laying their eggs in the larvae of the fruit fly *Drosophila melanogaster*. However, when wasps and flies cohabit, the flies protect their future offspring by choosing to lay their eggs in substrates that contain ethanol, as this keeps the wasps away (Kacsoh et al., 2013). Mothers keep on showing this shift in egg-laying preference even after the wasps have left, but only if they can form long-term memories.

In certain circumstances, it could also be helpful for organisms to know what to do in a given situation without having experienced it before. Now, in eLife, Julianna Bozler, Balint Kacsoh and Giovanni Bosco report that, for five generations, the descendants of flies exposed to wasps also choose to lay their eggs in places that contain ethanol, even in the absence of wasps (Figure 1; Bozler et al., 2019).

**Figure 1.**
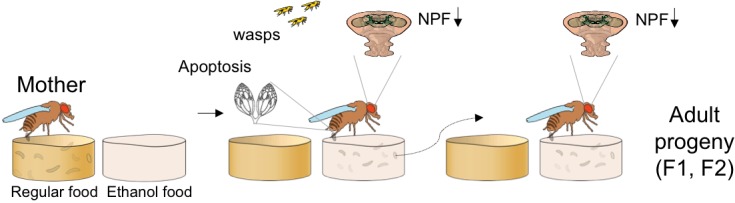
Transgenerational inheritance of egg-laying preference in flies. In normal conditions (left panel) *Drosophila* females prefer to lay their eggs in food that does not contain ethanol. However, when they cohabit with endoparasitoid wasps, they start to prefer to lay their eggs in food that contains ethanol, as this protects against the parasite. This switch in behavior is accompanied by decreased expression of neuropeptide F (NPF) in the brain and germcell apoptosis (middle panel). The adults hatched from these eggs (right panel) also prefer to lay eggs in food with ethanol and have reduced NPF levels in the brain, even though they have never been exposed to the wasps. The behavioral switch is maintained for up to five generations (not shown). F1: First filial generation. F2: Second filial generation.

This transmission of ethanol preference over several generations has a number of distinctive features. First, the maternal ancestor does not need to form a memory of the wasp exposure for the descendants to prefer ethanol-containing food as an egg-laying substrate. Second, the transmission only occurs in broods laid immediately after wasp exposure. Finally, if after five generations the descendants are not re-exposed to wasps, they lose their preference for ethanol and return to their normal egg-laying behavior.

While it is clear how this switch towards ethanol may benefit the flies, it could mean extinction for the wasps – that is, unless the parasites increase their ethanol tolerance or look for different hosts. And indeed, more specialist wasps that strongly depend on *Drosophila* as a host show a higher tolerance to ethanol, suggesting that they have evolved in response to this particular defense mechanism (Bouletreau and David, 1981; Milan et al., 2012).

To look into how this temporary inheritance works, Bozler et al., who are based at the Geisel School of Medicine at Dartmouth, first harnessed mRNA sequencing tools to examine the heads of parents and offspring. This did not reveal any obvious molecular signature that could help to understand the mechanism at work. However, they found that, in mothers, wasp exposure triggers germline apoptosis, a process in which germcells go through programmed death. In the heads of both mothers and offspring, there was also a reduction in the levels of neuropeptide F (NPF), a protein that controls food intake, alcohol preference and mating behavior in a number of organisms (reviewed by Nässel and Wegener, 2011). Further experiments using classical genetic approaches revealed that descendants needed to inherit the maternal *NPF* locus so that ethanol preference could be present in at least two generations. Taken together, these results suggest that the *NPF* gene is repressed after exposure to wasps: this downregulation would then pass on from mother to offspring, and influence their behavior accordingly.

Many aspects of this mechanism are still left to explore; for instance, it is unknown whether germline apoptosis and the reduction of NPF are independent events or somehow connected. Similarly, how the maternal NPF gene is repressed in response to wasp exposure, how this is transmitted to the offspring, and how this mechanism influences egg-laying preferences all remain unclear.

Changes in BDNF, another broad neuromodulatory pathway, also alter patterns of alcohol response across generations of mice (Finegersh and Homanics, 2014). If BDNF and NPF, which both mediate many behavioral performances, dictate transgenerational inheritance, this may suggest that mothers may unintentionally pass on more than just ethanol preferences.

The observations by Bozler et al. are not an isolated event. In many organisms, including humans, exposing parents to harsh conditions (such as food deprivation) has a lasting impact on descendants over several generations (reviewed by Jawaid and Mansuy, 2019). Once the conditions become favorable again, the behavioral changes disappear. Centuries ago, Lamarck proposed that creatures could pass on characters acquired during their lifetime, and the results from Bozler, Kacsoh and Bosco now shed an intriguing mechanistic and conceptual light on this controversial idea.

Inheriting a behavior involves two different components: a mature nervous system, and pluripotent germ cells. It is not yet known if experience independently modulates these two components, or if the nervous system ‘talks’ to the germ cells – and, if it does, it is not clear how experience-specific information is imprinted onto the newly formed nervous system. A number of possible mechanisms have been proposed, such as non-coding RNA, microRNA, DNA methylation, and histone modification, but the neuronal and molecular basis of this fascinating problem remains elusive (reviewed in Heard and Martienssen, 2014). Further exploring how egg-laying behavior is transmitted in *Drosophila* may yield important mechanistic insights into this question.
